# Sustainable biopolymer soil stabilization in saline rich, arid conditions: a ‘micro to macro’ approach

**DOI:** 10.1038/s41598-022-06374-6

**Published:** 2022-02-21

**Authors:** Samuel J. Armistead, Colin C. Smith, Sarah S. Staniland

**Affiliations:** 1 Department of Chemistry, The University of Sheffield, Dainton Building, Brook Hill, Sheffield, S3 7HF UK; 2 Department of Civil and Structural Engineering, The University of Sheffield, Sir Frederick Mappin Building, Sheffield, S1 3JD UK

**Keywords:** Chemical biology, Biogeochemistry, Environmental sciences, Natural hazards, Solid Earth sciences, Chemistry, Engineering

## Abstract

Water scarcity in semi-arid/arid regions is driving the use of salt water in mining operations. A consequence of this shift, is the potentially unheeded effect upon Mine Tailing (MT) management. With existing stabilization/solidification methodologies exhibiting vulnerability to MT toxicity and salinity effects, it is essential to explore the scope for more environmentally durable sustainable alternatives under these conditions. Within this study we investigate the effects of salinity (NaCl, 0–2.5 M) and temperatures associated with arid regions (25 °C, 40 °C), on Locust Bean Gum (LB) biopolymer stabilization of MT exemplar and sand (control) soil systems. A cross-disciplinary ‘micro to macro’ pipeline is employed, from a Membrane Enabled Bio-mineral Affinity Screen (MEBAS), to Mineral Binding Characterisation (MBC), leading finally to Geotechnical Verification (GV). As predicted by higher Fe_2_O_3_ LB binding affinity in saline in the MEBAS studies, LB with 1.25 M NaCl, results in the greatest soil strength in the MT exemplar after 7 days of curing at 40 °C. Under these most challenging conditions for other soil strengthening systems, an overall UCS peak of 5033 kPa is achieved. MBC shows the critical and direct relationship between Fe_2_O_3_-LB in saltwater to be ‘high-affinity’ at the molecular level and ‘high-strength’ achieved at the geotechnical level. This is attributed to biopolymer binding group’s increased availability, with their ‘salting-in’ as NaCl concentrations rises to 1.25 M and then ‘salting-out’ at higher concentrations. This study highlights the potential of biopolymers as robust, sustainable, soil stabilization additives in challenging environments.

## Introduction

Stabilization/solidification is used by engineers to improve the geotechnical characteristics of soils for a range of purposes (slope stabilization, low carbon building materials, road/building foundations). Ordinary Portland Cement (OPC), is typically added to soils in high quantities (10% by weight), forming a homogenous, rigid microstructure, improving soil’s cohesive strength properties^1,2^. The ability of OPC to improve soils strength has led to its global and universal use for a huge range of building and geotechnical applications. Some of the largest geotechnical structures are Mine Tailings (MT) dams, where waste from mining activity is stored. These facilities are often gigantic in size, at their largest, reaching heights of over 100 m and storing over 1 billion m^3^ of waste, typically built up over decades of mining^3^. Using OPC to stabilises these vast structures presents very real environmental issues. Notably OPC accounts for 5–7% of anthropogenic carbon dioxide emissions with 1 tonne of cement = 1 tonne of CO_2_^4,5^. In its production, OPC further consumes huge quantities of resources, such as raw materials (limestone, clay and sand), water and energy^6,7^. This is accompanied by emissions of SOx, NOx, toxic particulate matter, carbon oxides, metals, hydrogen fluoride/chloride and carbon monoxide by cement production plants, resulting in numerous local and global detrimental environmental effects^8^. The scale of MT structures further compounds the environmental issues associated with OPC use^9^. Furthermore there is growing evidence that OPC does not offer optimum strengthening under the conditions MT dams operate, such as sulphate rich, acidic, saline and arid environments^10–13^.

Mining operations within arid/semi-arid regions account for up to 50% of major metal production (Ag, Au, Cu, Pb, Zn)^14^. Due to low rainfall quantities (25 mm to 500 mm average rainfall per year), high temperatures (up to 30–45 °C average) and the sheer size of mining operations, water scarcity has become a major factor for these activities, Chile being a key example^14–17^. In order to relieve water stresses, mining companies have increasingly looked towards the use of seawater. In coming years, it will therefore become critical that MT stabilization/solidification additives are able to withstand highly saline conditions, as saline operations become the new normal. When considering OPC stabilizations use within saline conditions, limitations are found^18^. Reactions between salt anions (Cl^−^) and cement matrices cause the formation of voluminous, Friedls salt compounds, resulting in the expansion and cracking of cements rigid microstructure, reducing soil strength over time^19,20^. The long term use of OPC, to prevent MT failures within saline environments, is therefore untenable.

A lack of understanding of the detrimental environmental effects of OPC, as well as the increased financial and environmental costs of using OPC at large scale, means more and more MT dams are under stabilised and ultimately unsafe. When MT dams fail, it leads to catastrophic humanitarian and environmental disaster^21^. At least one major MT disaster occurs each year, for example in 2019 a failure in Brumadinho, Brazil resulted in the release of 11.7 million m^3^ of mining mud, causing at least 220 deaths and devastating environmental damage to over 600 km of the Rio Paraopeba^22,23^. Therefore, as a global community, we critically requires a more sustainable, scalable alternative to OPC, that functions optimally for the range of challenging environments that mining facilities operate, with saline-arid conditions being a principle environment of interest.

Chemical^24^, physical^25^ and biological^26^ based approaches have been explored as sustainable OPC soil stabilization alternatives. Chemical and physical approaches however create their own environmental and effectivity concerns^27,28^. Biological based methodologies, such as microbial/enzyme induced cementation (MIC/EIC), have attracted increasing attention due to desirable qualities, such as; renewable, low carbon production, low toxicity and their increasing economic viability^29,30^. Microbial induced calcium carbonate precipitation, in particular, has shown its ability to increase soil strength in numerous studies^31^. However both microbial and enzymatic methods are severely affected by MT’s biologically hostile conditions, which is further exacerbated by increased temperatures and salinity^32–34^. Further exploration is therefore required to discover a more environmentally durable solution, whilst retaining the sustainability benefits biological stabilization offers.

Biopolymer*s* are polymers derived from natural sources. They can be divided into three major classifications; polynucleotides (e.g. DNA, RNA), polypeptides (e.g. proteins) and polysaccharides (e.g. carbohydrates). Our inspiration for using biopolymers in geotechnical engineering is: 1. low environmental impact; 2 sustainability; 3. non-toxicity and 4. low cost, typical of biological stabilization methodologies^35^. A number of reviews have highlighted their significant potential in construction and geotechnical soil based applications^36–38^. Polysaccharide additives in particular offer increased quality control (reproducibility) and chemical versatility. Their ex-situ production, either through exo-cultivation or chemical extraction, provides control over production, preparation and addition methodologies^39^. They are also the most abundant polymers on earth, synthesized to fulfil many different biological functions (energy storage, structural support, gelling agents), therefore offering a plethora of versatile chemical functionalities^40^. Polysaccharide additives have also been shown to exhibit a high stability when exposed to saline conditions, across a broad pH range and when stored for long periods of time^41^.

Armistead et al.^42^ presents a ‘micro to macro’ MEBAS-MBC-GV methodological pipeline to assist in the rapid development of biopolymer soil stabilization additives. At the microscale, Membrane Enabled Bio-mineral Affinity Screen (MEBAS) is a fast method to assess a large number of bio-mineral binding interactions. Mineral Binding Characterisation (MBC) can then be performed on a smaller number of candidates to help understand the micro-scale mechanism. Translating to the macroscale-Geotechnical Verification (GV) demonstrates how bio-mineral interactions at the molecular level result in macroscopic geotechnical soil property improvements.

This pipeline has shown the capability to identify ‘high-affinity, high-strength’ bio-mineral composites, showing how this binding affinity at the microscale directly translates into highly increased unconfined compressional strength (UCS) of the soil at the macroscale. MEBAS assessment is ~ 50 fold quicker, when compared to a typical trial and error ‘top down’ methodology^42^. The ability to rapidly identify, understand and verify suitable biopolymer additives will dramatically catalyses progression in the field.

**Figure 1:**
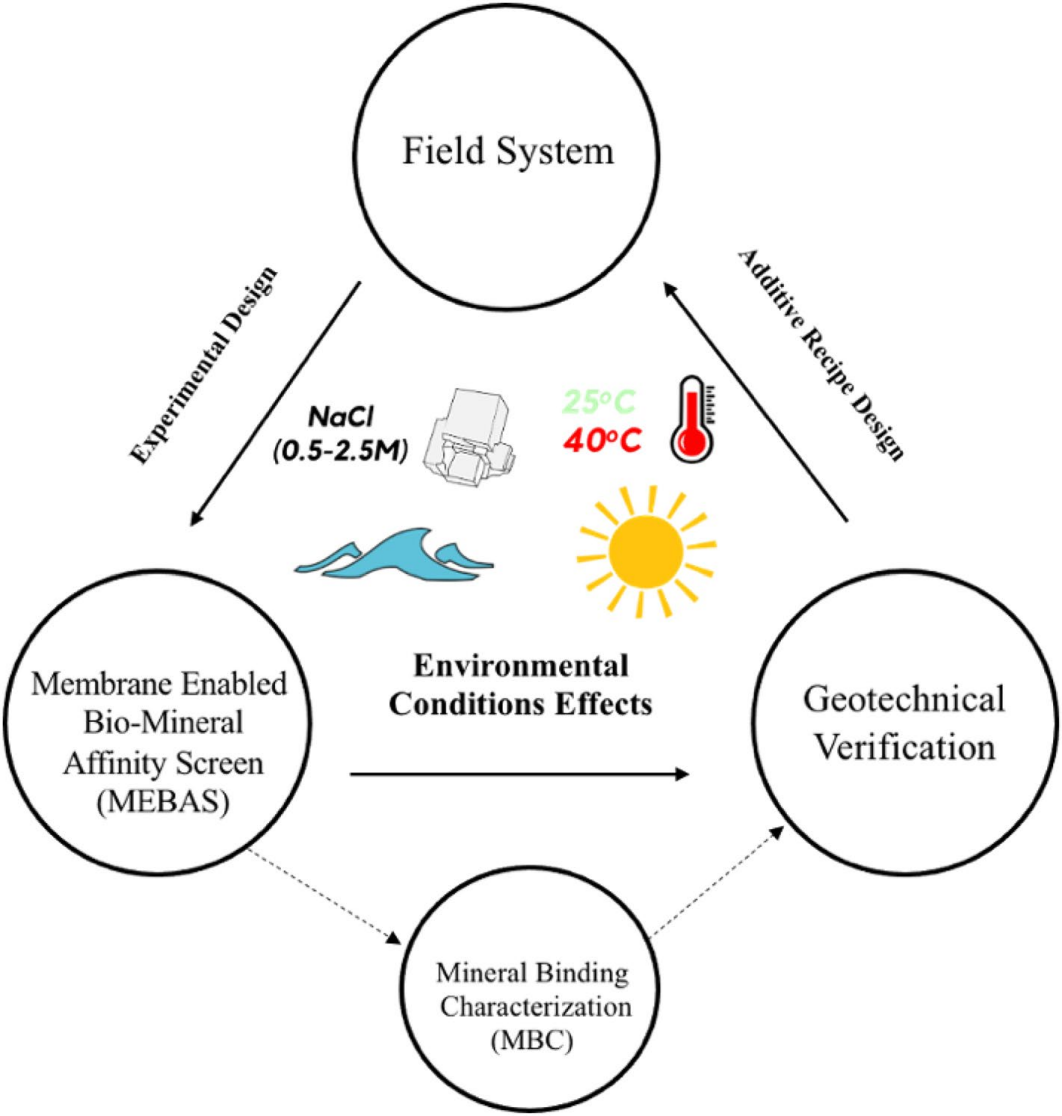
A schematic representing the MEBAS-MBC-GV methodological pipeline for the determination of environmental conditions effects upon a field system, through the micro (MEBAS-MBC) and macro-scales (GV), allowing the rapid design of specialised biopolymer additive recipes.

Here we build on previous research, utilizing the MEBAS-MBC-GV pipeline to investigate the effect of environmental conditions (salinity and temperature) through the micro and macro-scales (Fig. 1). Previously, the biopolymer Locust Bean Gum (LB) was found to vastly strengthen a MT exemplar soil matrix (sand containing 10% Fe_2_O_3_)^42^. Here we expand this work to test the applicability of this biopolymer in a range of environments including in saline (0.5–2.5 M NaCl) and arid (25 °C, 40 °C) mining conditions, emulating environmental conditions found for many MT facilities operating in water scarce regions. This study highlights the potential of biopolymer additives as next generation, sustainable geotechnical solutions.

## Results

### Field system selection

In this study, the effect of salinity/aridity on biopolymer stabilized soil properties has been investigated through the micro and macro-scales, utilizing MEBAS-MBC-GV methodology optimized in our previous study^42^. Discussion of the experimental design for the MEBAS-MBC-GV pipeline, as well as alignment of trends through the pipeline and potential avenues of exploration, can be found within Armistead et al.^42^. Fe_2_O_3_ (abbreviated to Fe in sample names and henceforth in the text) was selected as the mineral of focus, due to its universal and consistent abundance in MT, and the relative activity of iron minerals within fresh MT material^42^. Furthermore, surprisingly, little research has focussed on utilizing abundant Fe minerals for soil stabilization mechanisms. Therefore micro-scale MEBAS-MBC experiments have been used to investigate Fe-biopolymer affinities under different conditions.

In macro-scale GV experiments, a simplified MT exemplar soil system (abbreviated to MT in sample names) composed of Fe_2_O_3_ (10% by weight, 224 nm ± 119 nm) and SiO_2_ (90% by weight, 90–150 μm) (representing relatively inactive gangue material) was constructed to emulate the chemical composition (Supplementary Table S1) and particle size distribution^43^ found for real MT systems. No soil organic matter was added, due to fresh MT material typically containing negligible organic contents^44^. A pure sand (100% SiO_2_) mineral control (abbreviated to C in sample names) system is also investigated for comparison.

LB was selected for this study following the identification of its ‘high-affinity, high-strength’ Fe_2_O_3_ interactions^42^. LB was optimised through preliminary investigations (1% Mass_biopolymer_/Mass_soil_, 27.5% Mass_biopolymer_/Mass_water_, 0.2 M (Supplementary S2))^42^. Environmental conditions were selected to simulate field conditions found in water scarce regions (arid), increasingly utilizing sea water (saline) within operations^15^. As sea water contains a NaCl (abbreviated to S in sample names) concentration of 0.5 M, a concentration range of 0.5–2.5 M was selected due to the likelihood of elevated saline concentrations upon evaporation^45^. For micro-scale experiments, all concentrations were diluted to avoid biopolymer viscosity effects. In order to keep the experiments equivalent, the ratio of LB:S was kept the same (see “Materials and methods”)^42^. Micro-scale sample names have the addition of equivalent (eq.) to make this clear (e.g. S0.5–S2.5 eq.). The GV experiments are cured at two different temperatures (25 and 40 °C, abbreviated to T25 and T40 in the sample names) to assess engineering properties within field equivalent conditions. Low humidity effects (e.g. suction), typically found within arid environments, were not considered as a parameter under investigation, due to their previously identified negligible effect on strength improvements under comparable curing conditions^42^. The strength of non-stabilised (no LB), saline soils were investigated within medium saline (S1.25) concentration conditions. The list of sample names and their conditions are shown in Table 1.

**Table 1:**
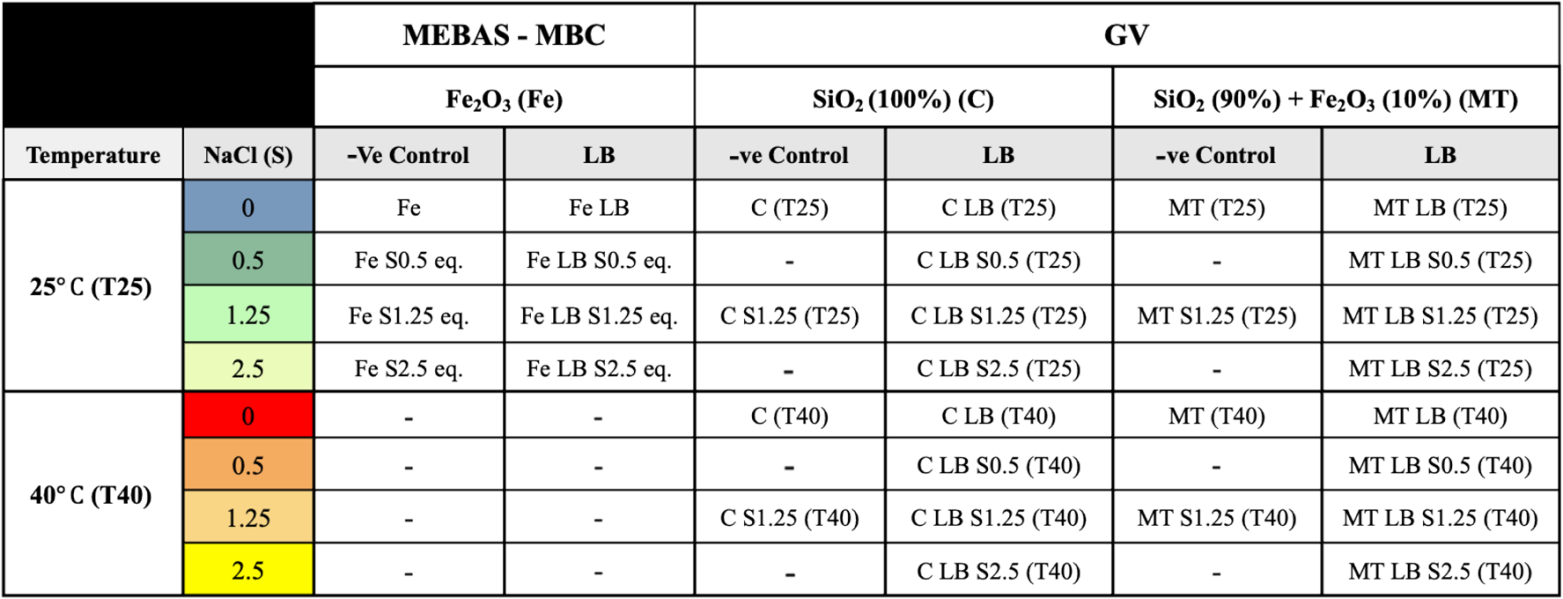
MEBAS-MBC-GV sample labels throughout the study.

### MEBAS of Fe LB binding affinity in saline conditions

**Figure 2:**
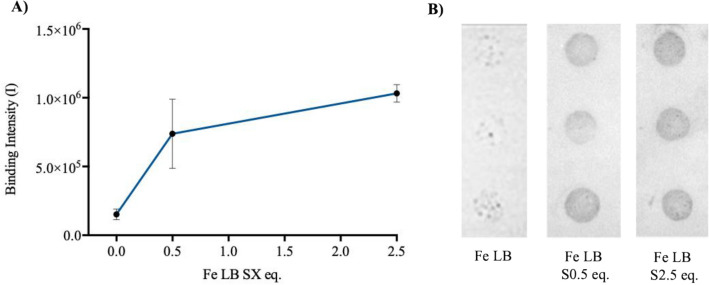
Membrane Enabled Bio-mineral Affinity Screen (MEBAS) analysis of Fe LB binding within saline conditions (S0–2.5 eq.), at pH 7. (**A**) Graph showing Fe LB binding intensities (Av+/− SD, in triplicate) upon a salinity gradient (S0–2.5 eq.). (**B**) Bio-Rad Chemi Doc membrane images of Fe LB within saline conditions (S0–S2.5 eq.).

In order to rapidly probe the bio-mineral binding intensities at the micro-scale, MEBAS was employed (Fig. 2). For control experiments, LB exhibits an affinity to Fe particles, as previously identified^42^. Upon the addition of S0.5 eq. a significant increase in Fe LB binding intensity was observed (Fig. 2A). When increasing salinity concentration (S0.5–2.5 eq.), further increases in binding intensity were observed. Figure 2B shows homogenous binding at all salt concentrations (S0.5–2.5 eq.), indicating the increased availability of Fe binding groups on the LB.

### MBC of Fe LB in saline conditions

**Figure 3:**
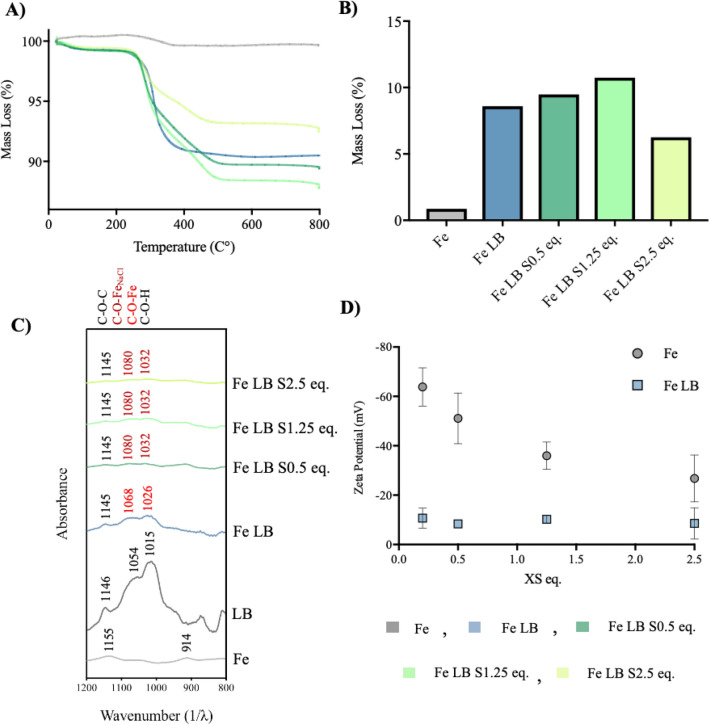
Mineral Binding Characterization (MBC) of Fe LB coated particles within saline conditions (S0–2.5 eq.) at pH 7 (**A**) TGA–mass loss curves upon a temperature change. (**B**) TGA–total mass loss upon a temperature change (200–600 °C). (**C**) ATR-FTIR of major region of interest (C–O–H Bending). (**D**) Zeta potential of Fe and Fe LB particles.

MBC has been used to understand the changes in bio-mineral binding intensities (Fig. 3). Thermal Gravimetric Analysis (TGA) was used to determine the quantity of organic coating on the mineral particle surface at different salinities, through combustion of the organic surface species. TGA thermal gradient profiles show the temperature at which particle coatings degrade, with the mass-loss directly attributed to the amount of biopolymer bound to the Fe surface (Fig. 3A)^46,47^.

The addition of salt shows a clear variation in the TGA profile, with a significant increase in mass lost observed up to S1.25 Eq. (10.746%) (Fig. 3B). When further increasing salinity (S2.5 eq.) a significant decline in mass loss was seen (10.746% to 6.256%), showing less coating, indicating less affinity at this higher salt concentration.

In this study Attenuated Total Reflection (ATR)–Fourier Transform Infra-Red (FTIR) was used to probe the effects of salinity on Fe LB chemical interactions associated with the adsorption of LB molecules at the bio-mineral interface (Fig. 3C)^48^. When LB molecules bind to the Fe surface, LB C–O–H absorption bands exhibit a wavenumber shift (1019 cm^−1^ to 1026 cm^−1^ and 1054 cm^−1^ to 1068 cm^−1^) and an absorbance intensity drop, associated with formation of C-O-Fe bonds.

When introducing salt (S0.5–2.5 eq.) further reductions in absorption intensity and peak shifts were observed (1026 cm^−1^ to 1032 cm^−1^ and 1068 cm^−1^ to 1080 cm^−1^), indicating the further conversion of C–O–H groups to C–O–Fe.

Zeta potential is a technique used to characterize surface charge at the solid/liquid interface and can probe the effects of LB coating and salinity upon the Fe surface (Fig. 3D). The control sample (Fe) exhibits a negative charge of -63.81 mv. Upon addition of salt (S0.5–2.5 eq.), a reduction in negative surface charge was observed, with Fe S2.5 eq. exhibiting a charge of − 26.786 mv.

LB coated Fe particles (Fe LB) displayed a significant reduction in negative surface charge, relative to Fe control sample (Fe LB =  − 10.696 mv compared to Fe =  − 63.81 mv). In contrast to the uncoated particles (Fe S0.5 eq.–Fe S2.5 eq.), when introducing salt to the LB coated particles (Fe LB S0.5–2.5 eq.), no significant change in surface charge was observed, indicating little change in surface electrostatics (Fig. 3D).

### GV of LB stabilised soils under saline conditions

To assess how this increased binding of LB to Fe particles in saline conditions translates to overall soil strength, GV was performed using unconfined compression strength (UCS) tests, typically used for cohesive soils.

**Figure 4:**
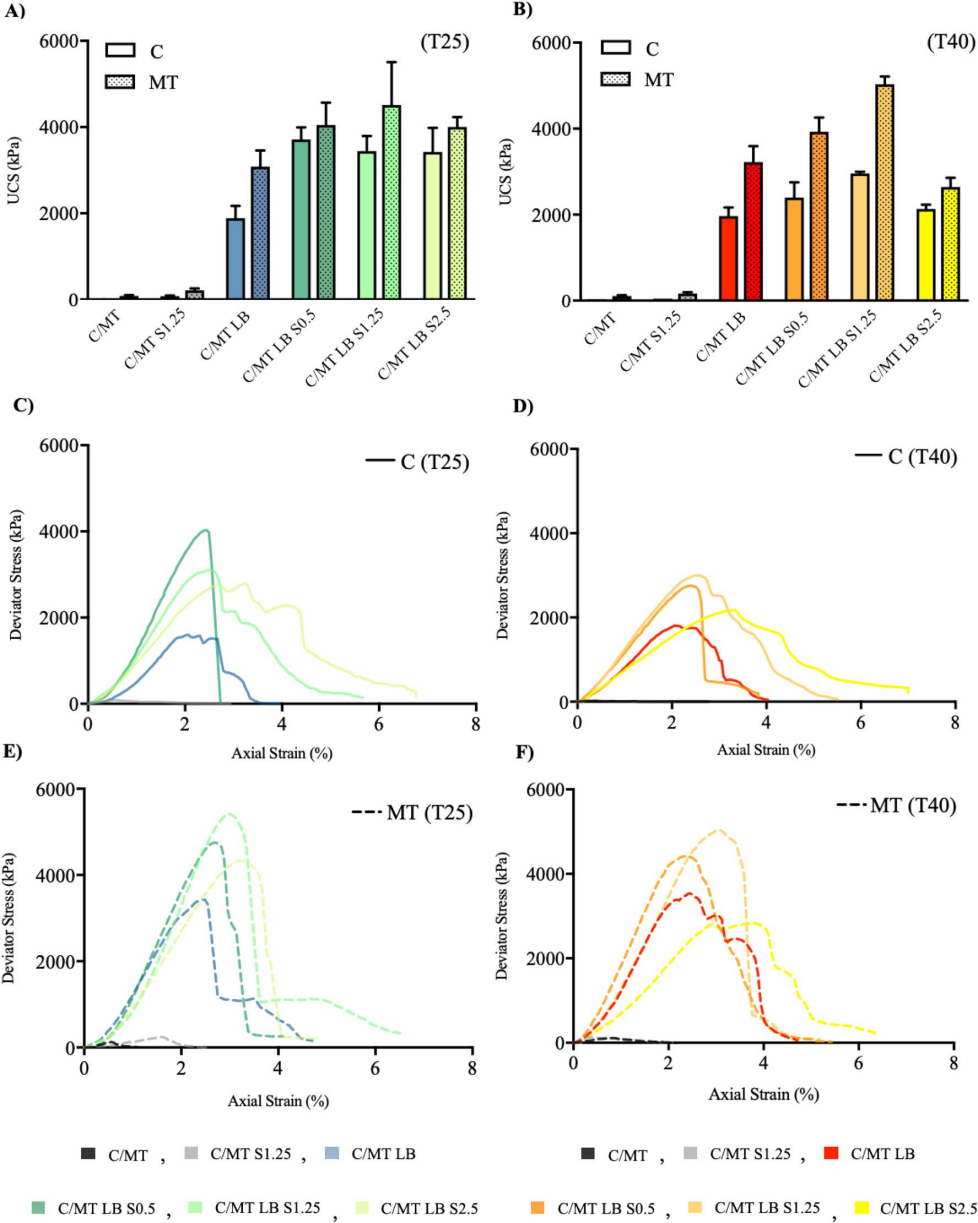
Geotechnical Verification (GV). Unconfined Compressional Strength (UCS) of samples (Av+/− SD, in triplicate) at (**A**) T25 (**B**) T40 (Tabulated UCS values can be found within Supplementary Table S3). Stress–strain profiles of C samples at (**C**) T25, (**D**) T40). Stress-stain profiles of MT samples at (**E**) T25, (**F**) T40.

Relative to mineralogical controls (C (T25)), the addition of LB resulted in a significant improvement in UCS, increasing from 0 to 1828 kPa from C (T25) to C LB (T25) (Fig. 4A). When salt was added, the UCS raises further, approximately doubling to 3713 kPa for C LB S0.5 (T25). A slight reduction in UCS (7–8%) was observed at higher salt concentrations (S1.25–2.5) (Fig. 4A).

For the MT soil system, the UCS of MT LB rises dramatically when compared to controls (increasing from 83 to 3083 kPa from MT (T25) to MT LB (T25)) and notably, the strength of MT LB (T25) is ~ 70% more than C LB (T25), attributed to the presence of Fe in the MT system, which microscale analysis has shown to have high affinity for LB. On the addition of salt, UCS rises, peaking S = 1.25 M, with an UCS of 4510 kPa for MT LB S1.25 (T25). Further increases in salinity (S2.5) resulted in soil strength loss (11%) (Fig. 4A).

When considering curing at the higher temperature of 40 °C, an equivalent trend to MT LB (T25) salt series was observed, with UCS peaking at 5032 kPa for 1.25 M salt (MT LB S1.25 (T40)) (Fig. 4B). While greater soil strength is achieved at this higher temperature, it is notable that the decrease in soil strength at S = 2.5 M is also more dramatic. There is a 48% loss of strength from MT LBS 1.25 (T40) to MT LB S2.5 (T40) compared to an 11% loss at 25 °C from MT LB S1.25 (T25) to MT LB S2.5 (T25) (Fig. 4B).

Interestingly, the C LB salt series show the same trend as the MT LB salt series at 40 °C with the greatest UCS at S = 1.25 M. This is in contrast to the trend seen for the C LB salt series at 25 °C. However, the C LB (T40) salt series exhibits reduced UCS improvements compared to the C LB (T25) salt series, with peak UCS of value of 2956 kPa for C LB S1.25 (T40), below 3000 kPa, while the C LB (T25) salt series all show values consistently above 3100 kPa (Fig. 4B).

For all C LB and MT LB samples, across both temperatures, on increasing salinity, an increased axial strain at peak strength is seen (Supplementary Figs. S4, S5). It is worth noting that a negligible correlation was observed between UCS and both moisture retention and void ratio for all LB stabilised samples (Supplementary Figs. S4, S5) indicating negligible contribution of suction and densification effects, relative to the biopolymer, for soil strength improvements.

## Discussion

When first considering mineralogical controls, MT and C show insubstantial strength characteristics (UCS = 0–109 kPa). This is attributed to negative surface charges of SiO_2_ (− 75 mv^49^) and Fe (− 63.81 mv, Fig. 3D), causing micro-scale particle repulsions and therefore a lack of cohesional strength on the macro-scale (Fig. 4). When 1.25 M of salt was added, for both temperatures (T25, T40), small increases in UCS (30–215 kPa) were seen (Fig. 4), ascribed to charge shielding effects of Na^+^, reducing negative repulsions (Fig. 3D) and increasing cohesive interactions, as previously reported^50^.

The salt series for all systems, with the exception of C LB (T25), displayed a trend of increasing UCS with increasing salt concentration up to a peak strength at 1.25 M, e.g. MT LB S1.25 (T25) (4510 kPa), MT LB S1.25 (T40) (5033 kPa) and C LB S1.25 (T40) (2956 kPa). For the MT series, MEBAS and MBC points to the strong affinity between the Fe surface and LB which is improved with salinity. An equivalent trend is seen in the TGA mass loss, with peak LB mass loss occurring at Fe LB S1.25 (Fig. 3B). FTIR provides evidence of LB binding to Fe through C-O-H groups, initially upon the addition of Fe and furthermore upon increasing salinity (Fig. 3C). High concentrations of NaCl (S 2.5 M eq.) likely prevents a portion of Fe LB containing C-O-H groups, as they are ‘salted-out’ and then removed during washing stages. Zeta potential shows that LB coating results in a significant reduction in surface charge, to within the threshold of aggregation (Fig. 3D). Upon the introduction of salt (S0-2.5 eq.), no significant change in surface charge was observed, indicating little electrostatic contribution to increased Fe-LB affinity in saline conditions. These results highlight that strength improvements for MT LB (T25) and MT LB (T40) samples are due to a combination of covalent and electrostatic bio-mineral interactions, as identified by our previous investigation^42^. However, the further strength improvements observed under saline conditions are due exclusively to covalent based Fe LB bio-mineral interactions.

Previous studies have shown the micro-scale effects of salinity on non-ionic biopolymers such as LB, with low concentrations resulting in a ‘salting in’ effect^51^. The phenomena of ‘salting in’ is driven by the interaction of Na^+^ with hydrophobic hydroxyl groups, causing their increased polarisation and ability to be solvated by water molecules^52^. This results in the opening up of hydrophobic groups, increasing their ability to form high-affinity bio-mineral interactions (Supplementary Figs. S6).

The loss of strength observed at highly saline conditions (S = 2.5 M) is postulated to be due to a combination of chemical and physical effects^51,53^. Chemically, highly saline conditions have previously been observed to cause a ‘salting out’ effect upon non-ionic biopolymer such as LB^51^. ‘Salting out’ is an entropic effect caused by the ‘structure making’ characteristics of Na^+^ ions^54^. Upon addition in high concentrations, Na^+^ dehydrates non-ionic biopolymer molecules such as LB, causing the reduced availability of binding groups for ‘high-affinity, high-strength’ bio-mineral interactions (Supplementary Fig. S4), therefore lowering soil strengths.

Physical effects are also proposed to contribute to strength losses at higher saline concentrations. When examining soil samples a dramatic increase in efflorescence can be observed from S1.25 to S2.5 samples (Supplementary Fig. S7), likely driven by phase separation in highly saline conditions. Although efflorescence is primary a surface aesthetic issue, the precipitation of salt within soil pores, known as sub-florescence, can cause crystallisation pressure, resulting in micro-cracking. Sub-florescence increases in smaller pores due to lower supersaturation thresholds, which may account for a proportion of the increased strength losses observed in MT samples^53^. The precipitation of salt crystals is also likely to disrupt bio-mineral binding.

Both increasing salinity and temperature have had a negative effect on soil strength for calcium mineral stabilised soils, which has been a considerable challenge to developing a sustainable approach in arid environments. Higher salt^55^ concentration and temperature^56^ are responsible for nucleating smaller calcium mineral crystals, which result in weaker inter-particle connections between soil particles, and therefore a lower bearing capacity at the macro-scale. For example, Chen et al.^56^ found a 60% reduction in strength for calcium carbonate stabilised soils, on increasing curing temperatures from 25 to 40 $^\circ{\rm C}$, with a significant reduction in crystal size (15–20 $\mu$ m to 2–5 $\mu$ m). This negative impact is not seen in our study where we move away from calcium-based systems. In fact the reverse, a positive effect is seen for iron-oxide-biopolymer stabilised systems with salt and higher temperatures, further demonstrating the importance of expanding investigations beyond calcium based stabilization for saline-arid environments.

When considering the salt series of C LB (T25), peak UCS was observed for C LB S0.5 (T25) soil, with further increases in salinity resulting in small decreases (7–8%) in strength (Fig. 4A). Due to SiO_2_’s highly charged nature, strength improvements are likely due to formation of non-specific electrostatic based bio-mineral interactions^57,58^. Increases in UCS within saline conditions are therefore postulated to be due to bio-mineral Na^+^ charge shielding effects. The small decrease in strength observed at higher salinities is attributed to physical sub-florescence effects.

When increasing curing temperature from T25 to T40, a number of strength trends are observed. Firstly, there is no significant difference seen in UCS, as a result of changing the temperature, for both C LB and MT LB soil systems, highlighting little sensitivity to arid-like environmental conditions and further reinforcing biopolymers potential use within higher temperature climates^41^. However, marked differences are seen when salt is present, with a dramatic increase in UCS up to S1.25 and decrease for S2.5 for MT LB (T40) sample series compared to MT LB (T25) (Fig. 4A,B). The more pronounced increase up to S1.25 and larger loss in strength for MT LB S2.5 (T40) is attributed to the entropic origin of both positive ‘salting in’ and negative ‘salting-out’ effects, resulting in increased energetic favourability at higher temperatures.

When comparing C LB (T40), with C LB (T25) salt series, strength improvements are attenuated (Fig. 4B), with a UCS peak occurring for C LB S1.25 (T40). This phenomena is attributed to increasing SiO_2_ negative surface charge, driven by the promotion of surface silonal group formation at higher temperatures (Supplementary Fig. S8)^49^. Increased negative surface charge is postulated to result in the dilution of Na^+^ shielding effects and weaker electrostatic, bio-mineral interactions. Fe surface charges are unaffected by temperature increases^59^. These results highlight the varying effects of curing temperature conditions when considering covalent (Fe-LB) and electrostatic (SiO_2_-LB) based bio-mineral interactions.

This study has highlighted a number of impactful and novel findings. The extension of MEBAS-MBC, from identifying and understanding ‘high-affinity, high-strength’ mineral binding molecules, to here probing the environmental conditions of solution salinity at the bio-mineral interface, has further highlighted the power of the MEBAS-MBC-GV methodological pipeline. With a rate of assessment over 50 times that of ‘top-down’ investigations, MEBAS offers a simple and effective tool for investigating how environmental conditions impact biopolymer additive use within a given soil system, before costly (time and resource), geotechnical investigations^42^. Notably, the correlation between bio-mineral affinities at the micro-scale (MEBAS-MBC) and macro-scale (GV) has been shown, further supporting the finding that a high-throughput, cross-disciplinary approach can be employed to decode highly heterogenous geotechnical soil systems.

This study has further reinforced the capacity of MBC to provide important micro-scale information, helping to understand the chemical bio-mineral driving forces at the molecular level that result in significant soil strength improvements. This understanding will allow for the better design and optimisation of biopolymer additives in future studies.

Critically, this study has shown that unlike OPC and other biological stabilization methodologies, where salinity and aridity cause detrimental effects, largely beneficial effect is seen with LB biopolymers. Further investigation will attempt to confirm that these effects are retained in the long-term and in real MT systems. Moreover, further studies will seek to show that the protected effects of bio-mineral associations to typical degradation pathways (hydrolysis, biodegradation) presents biopolymer additives as a long-term, durable, stabilization/solidification solution^60,61^.

This demonstrated promising ‘saline compatibility’ of LB (and potentially other biopolymer additives) indicate their potential future use to stabilise mining operations that utilize sea water where fresh water is scarce. It is noteworthy that globally 1 billion hectares of soils have high salt concentrations, accounting for 7% of all land, showing applicability in these soils too^62,63^. Further adoption of the powerful, systematic, MEBAS-MBC-GV methodological pipeline, to investigate more diverse soil systems, should catalyse progression within the field, unlocking the potential of biopolymers as next generation sustainable, chemical versatility and environmental durable OPC alternatives.

## Materials and methods

### Materials and reagents

Locust Bean Gum (LB), NaCl (S) and Ethylenediaminetetraacetic acid (EDTA) was purchased from Sigma Aldrich/Merck and used without further purification. Sand (SiO_2_) Fraction E (90–150 μm) was purchased from David Ball sand specialists. Fe_2_O_3_ (Hematite) was acquired from Mineral Waters Ltd and used as supplied.

### Biopolymer solution preparation

All LB solutions were prepared using the same methodology^42^. Powdered LB (MEBAS: 0.04 M, MBC: 0.01 M, GV: 0.2 M, Supplementary Fig. S2) was first added to temperature controlled (40 °C) ultrapure water and salt (S = MEBAS: 0.1–0.5 M, MBC: 0.025–0.125 M, GV: 0.5–2.5 M) solutions, whilst simultaneously agitating with a magnetic stirrer (micro: 600 rpm, macro: 300 rpm). Solutions were then incubated (10 min, 40 °C) and subsequently sonicated (10 min) using a VWR Ultrasonic water bath^42^. Concentration dilution was required when going from the macro-scale to the micro-scale to avoid biopolymer viscosity effects^42^. LB:S concentrations were maintained throughout to ensure macro and micro experiments were equivalent. Within each experimental section, concentrations prepared and their macro-scale equivalent conditions, which they are referred to throughout the study, are clearly outlined.

### Membrane enabled bio-mineral affinity screen (MEBAS)

MEBAS was carried out using nitrocellulose (0.2 $\mu$m pore size) membranes. 5 $\mu l$ drops of each prepared LB (0.04 M) solution was arrayed onto a nitrocellulose membrane in triplicate. After air drying, the membrane was submerged in a 3% wt/vol bovine serum albumin solution for 1 h to saturate any available nitrocellulose not covered by the arrayed LB. The membrane was then washed with ultrapure water before being subjected to an EDTA 10 mM wash for 1 h to ensure any bound metal cations are removed.

Binding experiment solutions were then prepared (500 ml, S0–aqueous solution, S0.1–2.922 g NaCl, S0.5–14.610 g NaCl). As previously outlined (biopolymer solution preparation), since LB:S is equivalent to macro-scale solutions, S0.1–S0.5 are referred to as S0.5–S2.5 eq. throughout this study. The pH was then adjusted to pH 7 using NH_4_OH (0.5 M)/HCl (0.5 M) where necessary. Membranes were then subjected to a final wash using binding experiment solutions (50 ml, S0-2.5 eq., 5 times, 2 min each). Fe (3 mg) particles were added to a solutions (50 ml). The resultant Fe suspensions were then sonicated (2 min, 8 kHz, 50:10 impulses). The prepared membrane was then submerged in the suspension for 4 h whilst rotating using a rotating using a Lab net Mini Labroller™. Following binding experiments, membranes were washed with fresh binding experiment solution (50 ml, S0-2.5 eq., 2 times, 10 min each). A Chemi-Doc gel documentation system (Bio-Rad, UK) was used to visualize and photograph the membranes and quantify the Fe binding intensities via densitometry.

### Mineral binding characterization (MBC)

LB (0.01 M) solutions were first prepared (20 ml, S0–aqueous solution, S0.025–0.029 g NaCl, S0.0625–0.073 g NaCl, S0.125 − 0.146 g NaCl). As previously outlined (biopolymer solution preparation), since LB:S is equivalent to macro-scale solutions, S0.025–S0.125 are referred to as S0.5–S2.5 eq. throughout this study. Fe (0.02 M, 64 mg) particles were then added and dispersed via sonication (10 min, VWR Ultrasonic water bath).

The solution pH was then adjusted to pH 7 using NH_4_OH (0.5 M)/HCl (0.5 M) where necessary. The solutions were then rotated for 30 min using a Lab net Mini Labroller™. Coated particles were separated using centrifugation (4000 rpm, 10 min) and washed using ultrapure water and saline (S0.5–2.5 eq.) solutions to remove excess non-bound biopolymers (4 repeats). Particles were then left to dry at room temperature, ready for analysis.

The mass of the organic particle coatings were determined using a PerkinElmer Pyris 1 Thermal Gravimetric Analyzer. Dry Bio-Fe particles were exposed to a temperature range of 20–800 $^\circ{\rm C}$ under a 2/3 N_2_, 1/3 O_2_ atmosphere.

Surface functional groups were determined using a Perker Elmer Frontier Fourier Transform Infrared (FTIR) and Golden Gate Diamond Attenuated Total Reflection (ATR) spectrometer. Data collection and analysis was performed using Spectrum™ 10. Scans were made between 4000 cm^-1^ and 400 cm^-1^. Baseline correction was performed on all spectra.

Zeta potentials were determined using a Brookhaven BI-900AT. Fe and Fe LB particles were ground using a pestle and mortar and dispersed (0.01 mg/ml) via sonication (10 min, VWR Ultrasonic water bath) in control (S, 0.01 M) and saline (S0.5–2.5 eq.) solutions. Micro-scale saline concentrations were employed for zeta measurements, as reproducibility issues were identified in macro-scale conditions (Supplementary Fig. S9). This is attributed to the formation of polydisperse particle solutions, within highly saline conditions, due to Na^+^ charge shielding effects. Polydisperse solutions have previously been observed to cause the variation in zeta potential peaks and increased width at half heights, as seen within this study (Supplementary Fig. S9), due to different sized particles resulting in heterogenous charge densities and Brownian motion effects^64^. The solution pH was adjusted to pH 7 using NH_4_OH (0.5 M)/HCl (0.5 M), where necessary. Samples were scanned 5 times at 25 $^\circ{\rm C}$ and data analysed using Malvern ZetaPlus software.

### Geotechnical verification-sample preparation

GV preparation condition optimisation can be found within a previous investigation^42^. LB (0.2 M, 27.5% (Mass_water_/Mass_soil_), corresponding to 1% Mass_biopolymer_/Masss_soil_ addition) solutions, once prepared within saline (S0.5–2.5) and non-saline conditions, were immediately mixed with 160 g of material (MT and C) until a homogenous mix was achieved.

The resulting composite was then divided into 3 equal parts and compacted using a cylindrical drop hammer (2.1103 kg, 246 mm × 37 mm) via 10, 126 mm drops, within a 202 mm × 42 mm hollow cylindrical sample mold. Samples were then extruded and left to cure (7 days at 25/40 °C). All sample series were prepared and cured at the same time to ensure identical curing within each condition. 40 °C curing temperatures were maintained using a temperature controlled Wykeham Farrance drying oven. Sample dimensions and masses were recorded to determine moisture retention and final densities.

### Geotechnical verification- unconfined compressional strength testing

A digital Tri-test ELE was used to perform unconfined compressional strength tests following the ASTM D2166 standard method^26^. Load (N) and displacement (1.5 mm min^−1^) data were collected during tri-axial tests. Sample bedding errors were removed pre-data analysis. The UCS (kPa) at failure of each sample was determined as the peak applied axial load (N), per cross sectional area. Axial strain (%) at peak strength was determined as the sample vertical displacement (mm) at failure as a proportion of the original sample height.

## Supplementary Information

Supplementary Information.
