# Supplementary material for: Sustainable biopolymer soil stabilization in saline rich, arid conditions: a ‘micro to macro’ approach
Source: Sci Rep. 2022 Feb 21;12:2880. doi: 10.1038/s41598-022-06374-6 (PMC8861022; doi:10.1038/s41598-022-06374-6)
Supplement: Supplementary file 1 — Supplementary Information. [file 41598_2022_6374_MOESM1_ESM.pdf]

Supporting Information

Sustainable Biopolymer Soil Stabilization in Saline Rich, Arid Conditions – A 'Micro to Macro' Approach

Samuel J. Armistead,[1,2] Colin C. Smith,[2*]
Sarah S. Staniland[1*]

[1]Department of Chemistry, The University of Sheffield, Dainton building, Brook Hill, Sheffield S3 7HF, UK

[2]Department of Civil and Structural Engineering, The University of Sheffield, Sir Frederick Mappin Building, Mappin Street, Sheffield, S1 3JD, UK

* Corresponding authors: Correspondence should be sent to c.c.smith@sheffield.ac.uk and s.s.staniland@sheffield.ac.uk

S1. Table showing typical Mine Tailing (MT) ore, mineral composition and pH.

Mine Tailing Site Location	Ore	Fe_2O_3	Al_2O_3	SiO_2	CaO	pH	Reference
China (A)	Fe	14.37	0.8	82.26	0.57	-	[1]
China (B)	Fe	12.61	1.65	75.46	1.70	-	[2]
South Africa (A)	Au	11.7	14.17	58.12	7.5	8.0	[3]
South Africa (B)	Cu	8.07	12.06	65.5	5.91	8.5	[4]
Tanzania	Au	10.40	9.61	50.74	5.34	7.2-7.5	[5]

S2. Example method to determine biopolymer solution molality.

Molarity = $Mass_{biopolymer}$ / ($Volume_{water}$ * Molecular Weight)

Molecular Weight = Average Biopolymer Molecular Weight (Repeat Unit)

LB Average Biopolymer Molecular Weight

= 180.16 $gmol^{-1}$ * 5 / 5 = 180.16 $gmol^{-1}$

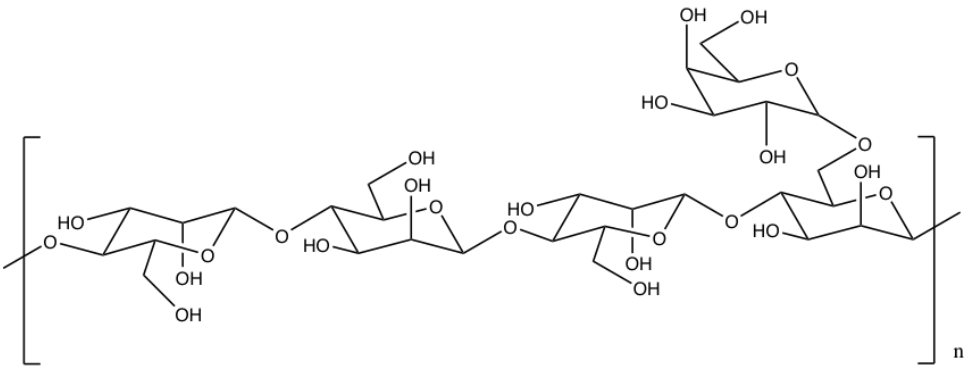

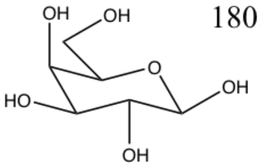

180.16 $gmol^{-1}$

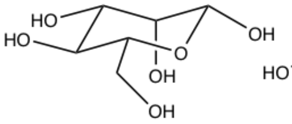

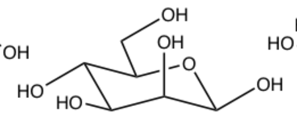

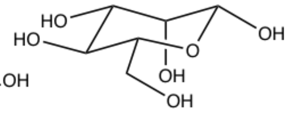

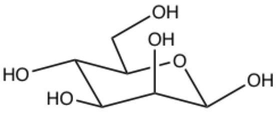

180.16 $gmol^{-1}$ 180.16 $gmol^{-1}$ 180.16 $gmol^{-1}$ 180.16 $gmol^{-1}$

S3. Table showing Geotechnical Verification (GV) macroscopic UCS (Av +/- SD, kPa) strength properties of C/MT LB S0-2.5 (T25/40) samples.

		Sample UCS (Av +/- SD, kPa)			
		SiO_2 (100%) (C)		SiO_2 (90%) + Fe_2O_3 (10%) (MT)	
**Temperature**	**NaCl (S)**	**-ve Control**	**LB**	**-ve Control**	**LB**
25°C (T25)	0	0	1828 +/- 278	0	3083 +/- 375
	0.5	-	3674 +/- 281	-	4045 +/- 522
	1.25	78 +/- 14	3347 +/- 333	215 +/- 41	4510 +/- 997
	2.5	-	3134 +/- 560	-	4002 +/- 228
40°C (T40)	0	18	1967 +/- 200	109 +/- 17	3223 +/- 368
	0.5	-	2399 +/- 353	-	3929 +/- 326
	1.25	29	2956 +/- 44	167 +/- 33	5033 +/- 181
	2.5	-	2133 +/- 99	-	2640 +/- 216

S4. Strength Characteristics of C/MT LB S0-2.5 (T25) Samples.

S4.1. Axial Strain at Peak Strength (%).

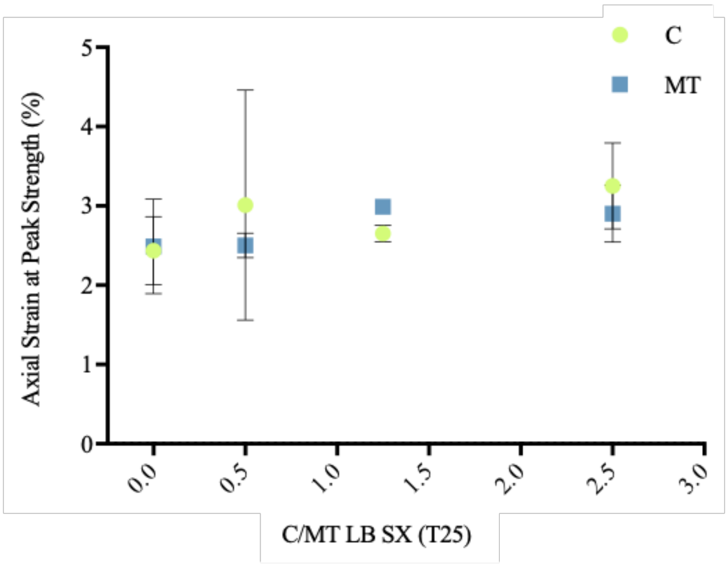

S4.2. Graphs showing the relationship between UCS and both moisture retention and void ratio of C/MT LB S0-2.5 (T25) samples. A) Relationship between moisture retention and UCS B) Relationship between void ratio (%) and UCS.

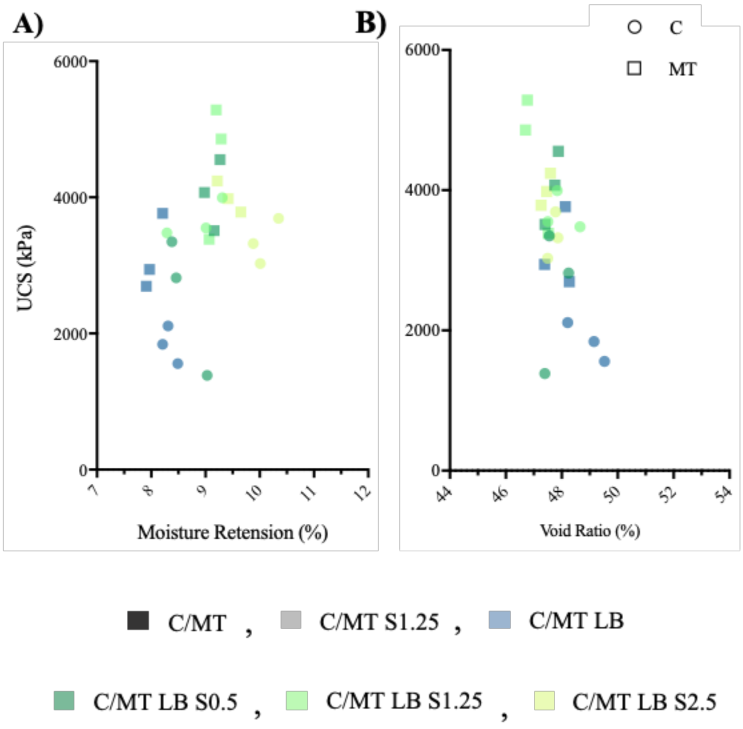

S5. Strength Characteristics of C/MT LB S0-2.5 (T40) Samples.

S5.1. Axial Strain at Peak Strength (%).

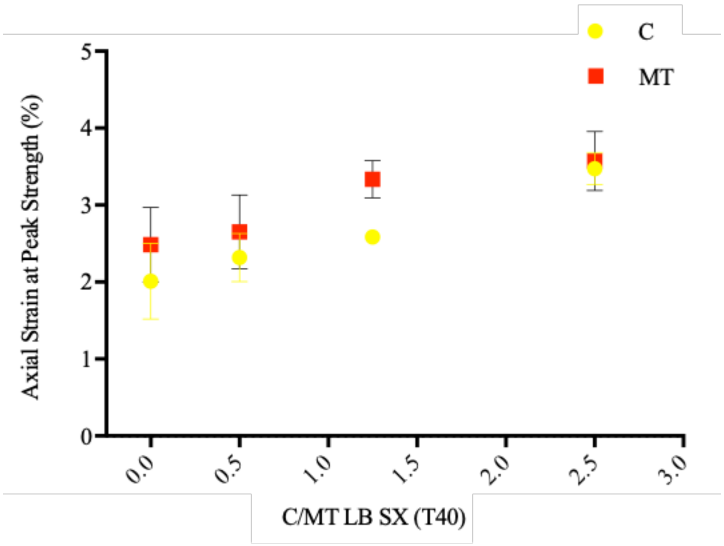

S5.2. Graphs showing the relationship between UCS and both moisture retention and void ratio of C/MT LB S0-2.5 (T40) samples. A) Relationship between moisture retention and UCS B) Relationship between void ratio (%) and UCS.

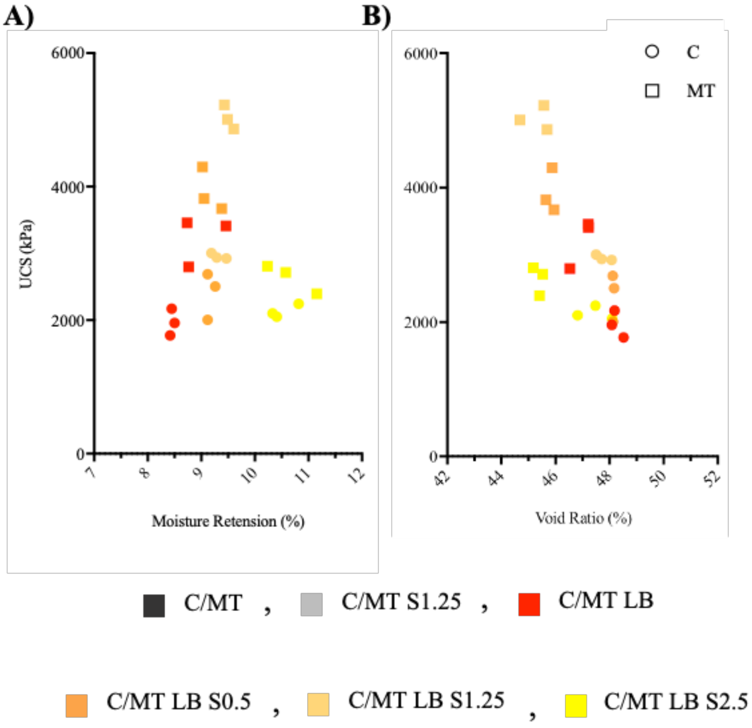

S6. Schematic representing the ‘salting-in, salting-out’ of LB hydrophobic mannose groups upon increasing salinity. Initial increases in salinity (S0-1.25) result in the ‘salting-in’ of mannose groups due to the increased polarisation of hydrophobic groups, increasing their ability to be solvated by water molecules. Upon further increases in salinity (S1.25-2.5) ‘salting-out’ occurs due to entropic factors driven by the high affinity of Na^{+} to water molecules. This results in the reduction of available hydroxyl groups for bio-mineral interactions.

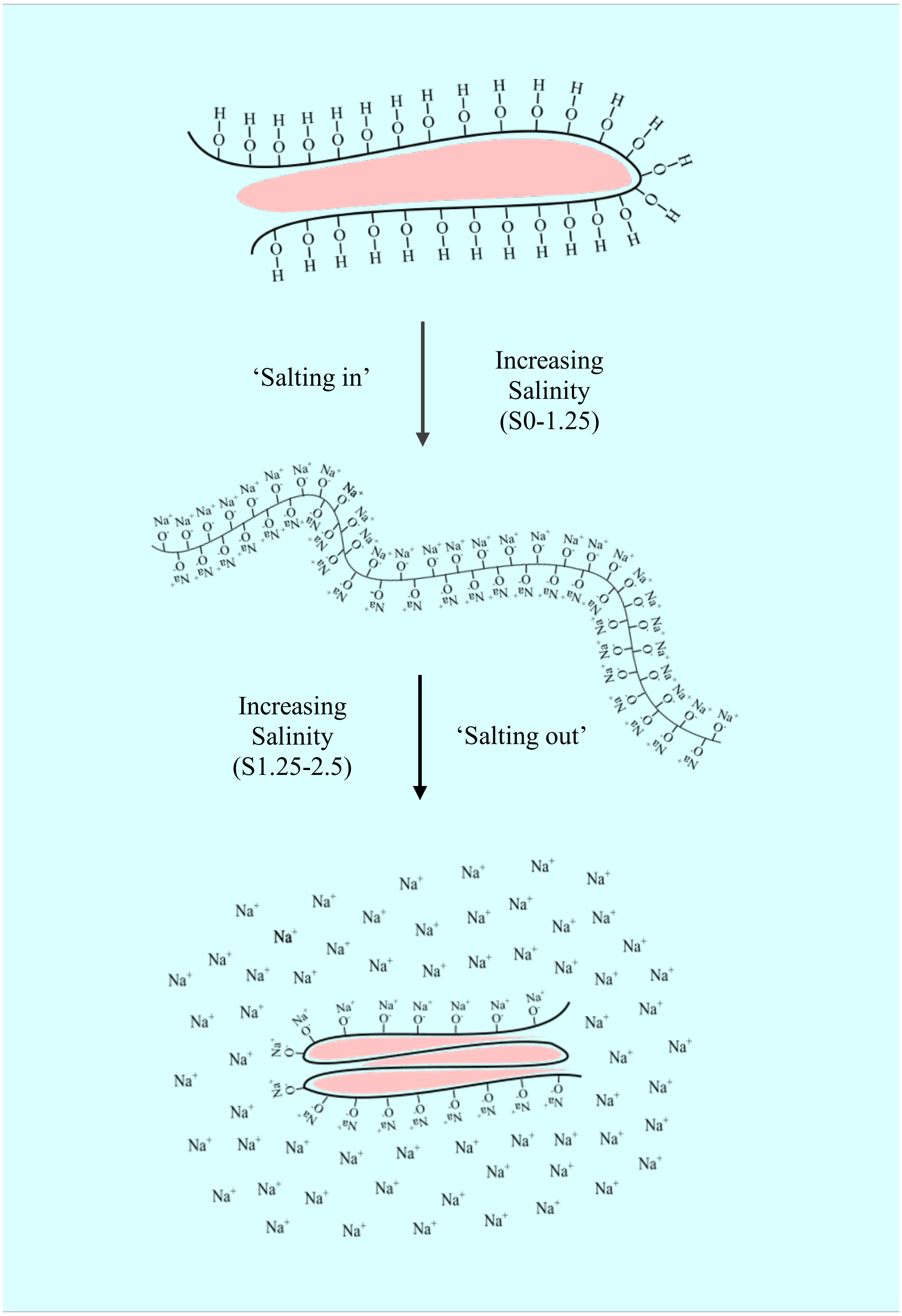

S7. Image highlighting the efflorescence of LB MT SX T25 samples. A) S1.25 B) S2.5.

A) **B)**

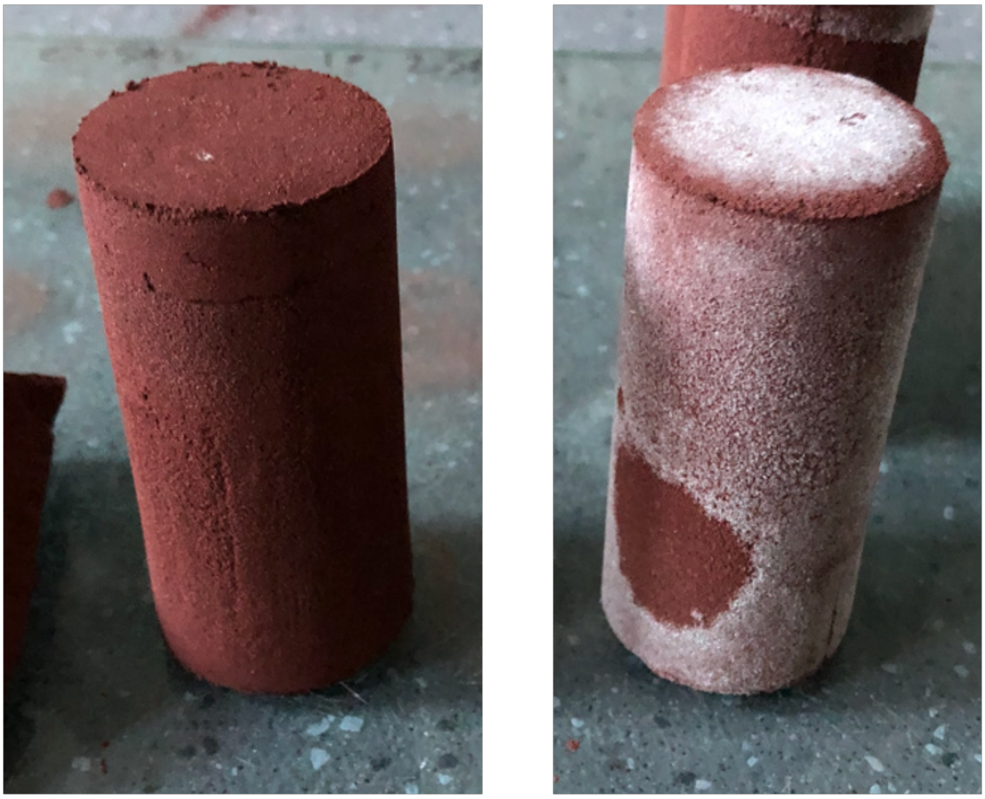

S8. Schematic representing increased SiO_2 negative charge density upon elevating temperature from 25℃ to 40℃, postulated to account for the reduction in UCS observed. Upon hydration, the formation of silonal groups is dependent upon temperature, with increased temperature resulting in increased silonal formation. As the UCS was performed at pH 7, silonal groups exist in their deprotonated form.[6]

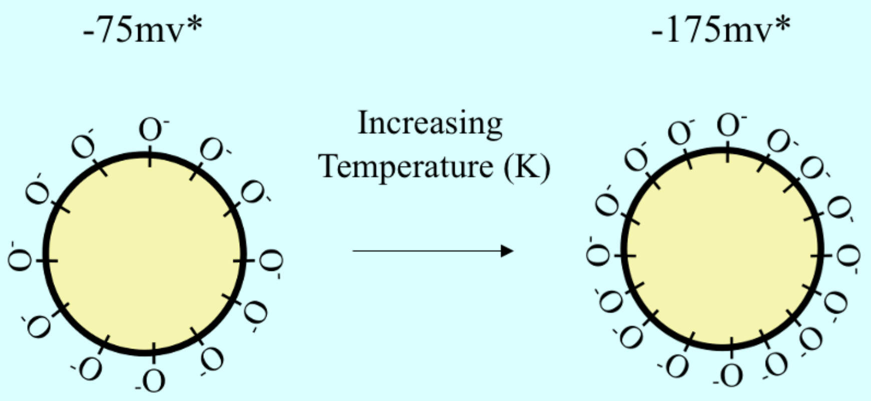

$$SiO_2 + 2H_2O = H_4SiO_4$$

$$\log (H_4SiO_4) = 0.151 - 1162/T$$

$$H_4SiO_4 = H^+ + H_3SiO_4^- \text{ , } pK = 9.8,$$

$$-9.8 = -\log (H_4SiO_4) + \log (H^+) + \log (H_3SiO_4^-).$$

S9. Saline condition effects (S0-2.5) upon the zeta potential of Fe (0.1 mg/ml) and Fe LB (0.1 mg/ml). A) Graph highlighting the zeta potential reproducibility issues associated with high S0.5-2.5 M concentrations. B) Graph showing the width at half height (mV) of Fe and Fe LB particles. Increased widths at half height associated with highly saline conditions (S0.5-2.5) are attributed to an increased particle size distribution.

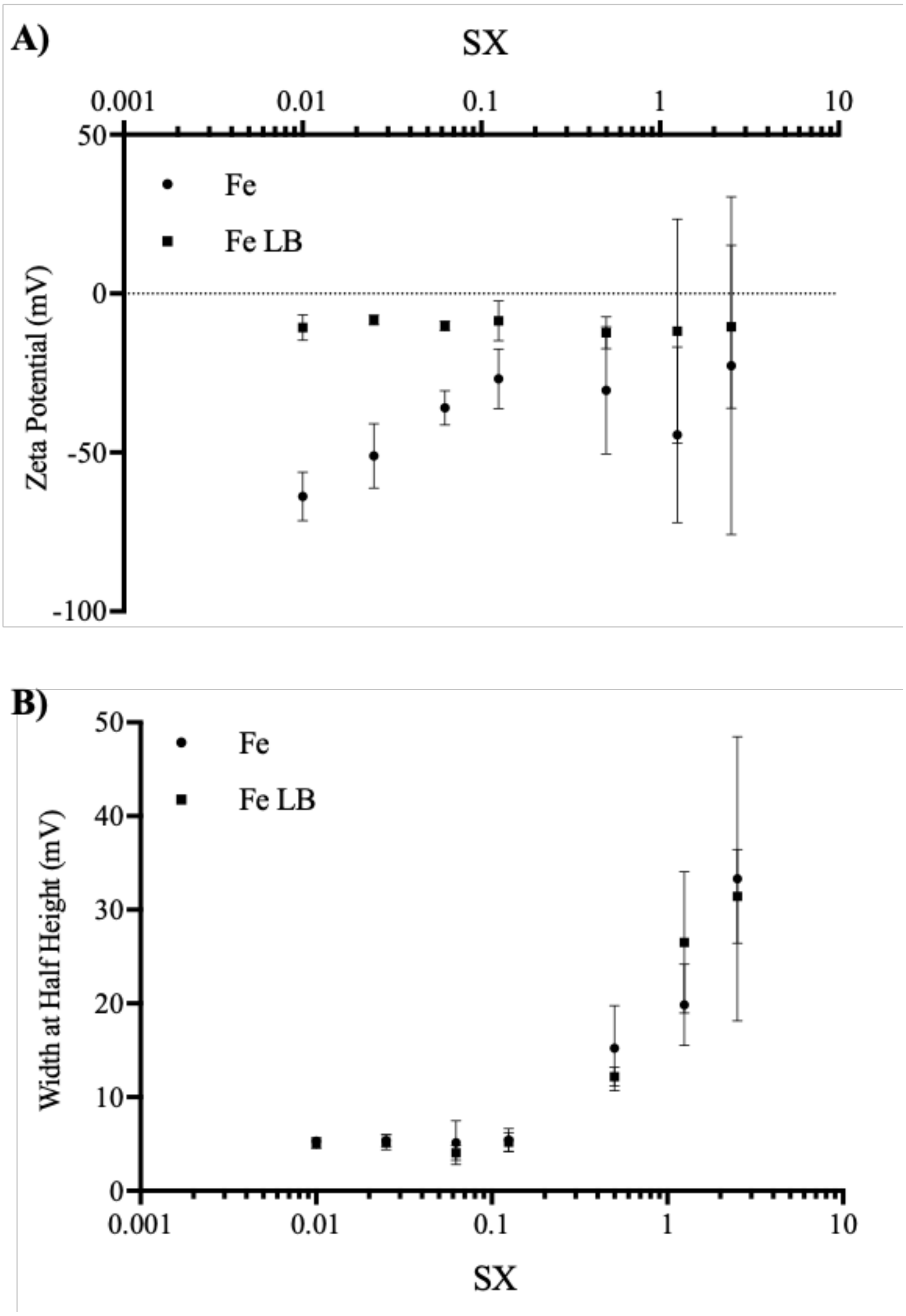

References

(1) Li, X.; Yu, H.; He, Y.; Xue, X. Synthesis of Fe-MCM-41 Using Iron Ore Tailings as the Silicon and Iron Source. *J. Anal. Methods Chem.* **2012**, *2012*, 1–5.

(2) Tang, C.; Li, K.; Ni, W.; Fan, D. Recovering Iron from Iron Ore Tailings and Preparing Concrete Composite Admixtures. *Minerals* **2019**, *9* (4), 232.

(3) Thobakgale, R.; Gitari, W. M.; Akinyemi, S. A. Evaluation of the Geochemical and Minerological Transofrmation at an Old Copper Mine Tailings Dump in Musina, Limpop Province, South Africe, University of Venda, 2017.

(4) Gitari, M. W.; Akinyemi, S. A.; Thobakgale, R.; Ngoejana, P. C.; Ramugondo, L.; Matidza, M.; Mhlongo, S. E.; Dacosta, F. A.; Nemapate, N. Physicochemical and Mineralogical Characterization of Musina Mine Copper and New Union Gold Mine Tailings: Implications for Fabrication of Beneficial Geopolymeric Construction Materials. *J. African Earth Sci.* **2018**, *137*, 218–228.

(5) R. P. Mapinduzi. Potential for Reuse of Gold Mine Tailings as Secondary Construction Materials and Phytoremediation. *Int. J. Environ. Sci.* **2016**, *7* (1), 49–61.

(6) Rodríguez, K.; Araujo, M. Temperature and Pressure Effects on Zeta Potential Values of Reservoir Minerals. *J. Colloid Interface Sci.* **2006**, *300* (2), 788–794.
